# An *Anopheles aquasalis* GATA factor Serpent is required for immunity against *Plasmodium* and bacteria

**DOI:** 10.1371/journal.pntd.0006785

**Published:** 2018-09-24

**Authors:** Ana C. Bahia, Marina S. Kubota, Jayme A. Souza-Neto, Leonardo B. Koerich, Ana Beatriz Barletta, Helena R. C. Araújo, Caroline M. Gonçalves, Cláudia M. Ríos-Velásquez, Paulo F. P. Pimenta, Yara M. Traub-Csekö

**Affiliations:** 1 Laboratório de Biologia Molecular de Parasitas e Vetores, Instituto Oswaldo Cruz, Fiocruz, Rio de Janeiro, RJ, Brazil; 2 Centro de Desenvolvimento Tecnológico em Saúde (CDTS), Instituto Oswaldo Cruz, Rio de Janeiro, RJ, Brazil; 3 Laboratório de Fisiologia de Insetos Hematófagos, Instituto de Biologia, Universidade Federal de Minas Gerais, Belo Horizonte, MG, Brazil; 4 Laboratório de Bioquímica de Artrópodes Hematófagos, Instituto de Bioquímica Médica Leopoldo De Meis, Universidade Federal do Rio de Janeiro, Rio de Janeiro, Brasil; 5 Laboratório de Entomologia Médica, Instituto René Rachou, Fiocruz, Belo Horizonte, MG, Brazil; 6 Laboratório de Ecologia de Doenças Transmissíveis na Amazônia, Instituto Leônidas & Maria Deane, Fiocruz, Manaus, AM, Brazil; 7 Fundação de Medicina Tropical Dr. Heitor Vieira Dourado, Manaus, Amazonas, Brazil; 8 Laboratório de Biodiversidade em Saúde, Centro de Pesquisa Leônidas & Maria Deane, Fiocruz, Manaus, AM, Brazil; University of Florida, UNITED STATES

## Abstract

Innate immunity is an ancient and conserved defense system that provides an early effective response against invaders. Many immune genes of *Anopheles* mosquitoes have been implicated in defense against a variety of pathogens, including plasmodia. Nevertheless, only recent work identified some immune genes of *Anopheles aquasalis* mosquitoes upon *P*. *vivax* infection. Among these was a GATA transcription factor gene, which is described here. This is an ortholog of GATA factor Serpent genes described in *Drosophila melanogaster* and *Anopheles gambiae*. Gene expression analyses showed an increase of GATA-Serpent mRNA in *P*. *vivax*-infected *A*. *aquasalis* and functional RNAi experiments identified this transcription factor as an important immune gene of *A*. *aquasalis* against both bacteria and *P*. *vivax*. Besides, we were able to identify an effect of GATA-Serpent knockdown on *A*. *aquasalis* hemocyte proliferation and differentiation. These findings expand our understanding of the poorly studied *A*. *aquasalis-P*. *vivax* interactions and uncover GATA-Serpent as a key player of the mosquito innate immune response.

## Author summary

*A*. *aquasalis* is an important natural malaria vector in the Americas, where most malaria cases are due to *P*. *vivax*. There is little information on both the insect and the parasite. One of the difficulties of working with *P*. *vivax* is the impossibility of culturing this parasite continuously, reason why our studies were performed using blood from malaria patients collected in the Amazon region. We have previously detected differences between the responses of *A*. *aquasalis* to *P*. *vivax* infection in relation to better studied mosquito-*Plasmodium* pairs, stressing the importance of studying different systems, since the development of new malaria transmission-blocking strategies may be insect/parasite-specific, and also have geographical specificities. In the present manuscript we demonstrate that the transcription factor GATA-Serpent is a key player in controlling parasite and bacteria development in these mosquitoes. Our data provide new insights into the GATA-Serpent-mediated mechanisms that regulate the *A*. *aquasalis* immune response against *P*. *vivax* and the first evidence of hemocytes involvement in this process.

## Introduction

Insects have a powerful innate immune system that includes hemolymph clotting cascades, melanin production, phagocytosis, encapsulation, antimicrobial peptide (AMP) synthesis and free-radicals production [1, 2]. Immune responses are typically orchestrated by molecular signaling cascades that lead to the activation of transcription factors (TFs). These TFs activate and regulate immune mechanisms by promoting the transcription of important genes and cellular responses to kill invaders. In anopheline mosquitoes some TFs have been described as key immune modulators. Among these are STAT, Rel1, Rel2 and Jun/Fos, which are part of the four main innate immune signaling pathways, JAK-STAT, Toll, Imd and JNK, respectively [3–7].

In the insect model *Drosophila melanogaster* TFs of the GATA family have been implicated in controlling hematopoiesis, early development and transcription of genes in immune tissues such as the fat body and the midgut [8–10]. GATA TFs have one or two zinc fingers that can bind to (AT) GATA (AG) sequences in the genome. They are conserved among fungi, plants, insects, and mammals, and are involved in cell proliferation and differentiation [11]. Most vertebrates have six GATA TFs divided in two classes, the GATA 123 and GATA 456. In *D*. *melanogaster* (Dmel) and *Anopheles gambiae* (Agam) five GATA factors are currently known. From these, only *DmelGATAc* (Grain) and *AgamGATA123* belong to the GATA 123 class; the other four [*DmelGATAa* (Pannier) and *Agam456bbb*, *DmelGATAb* (Serpent) and *AgamGATA456ba*, *DmelGATAd* and *AgamGATA456bbb*, and *DmelGATAe* and *AgamGATA456a*)] are GATA 456 insect orthologues [12]. In the *A*. *aquasalis* genome, we found three orthologues of GATA sequences: *Aaqu123* (Grain), *Aaqu456bbb* (Pannier) and *AaquGATA456ba* (Serpent). *Drosophila* haematopoiesis gives rise to three independent hemocyte lineages orchestrated by GATA and JAK-STAT immune signaling: plasmatocytes, crystal cells and lamellocytes. All these cells are involved in some aspects of insect immunity as melanization, phagocytosis, encapsulation and nodulation [1]. Mosquito hemocyte populations are dynamic and influenced by blood feeding and infection [13–17]. Three distinct mosquito hemocyte cells are identified in larva, pupa and adult based on morphological, antigenic, and functional markers: prohemocytes, oenocytoids and granulocytes [14]. In mosquitoes, the prohemocytes differentiate into both oenocytoids and granulocytes upon *Plasmodium* infection in a fashion that is dependent of the LPS-induced TNFα transcription factor-like 3 (LL3) and the JAK-STAT pathway [18]. We previously identified an *A*. *aquasalis* GATA factor Serpent gene by a subtraction approach and in this study we detailed the study of this gene (*Aaqu-Srp*). *A*. *aquasalis* was chosen for these studies due to its importance in *P*. *vivax* transmission at the Atlantic and Pacific coasts from Central America to southern Brazil and to the fact that it can be colonized and has been used in experimental infections and transmission assays with different *Plasmodium* species [19].

Here we examined *Aaqu-Srp* participation in the mosquito immune responses to bacteria and *Plasmodium vivax*, the main human malaria parasite in Brazil, which accounts for over 80% of infections in this country [20]. We found that GATA-Serpent is a key player in controlling parasite development in mosquitoes. We demonstrated that *Aaqu-Srp* is induced in the mosquito by *P*. *vivax* infection and that the knockdown of this gene results in increased bacteria and parasite loads, and interferes with hemocytes differentiation. Our data provide previously unidentified insights into the GATA-Serpent-mediated mechanisms that regulate the *A*. *aquasalis* immune response against *P*. *vivax* and the first evidence of hemocytes involvement in this process.

## Methods

### Mosquito feeding and maintenance

*A*. *aquasalis* (Curry, 1932) were reared in an insectary at 28°C, 80% humidity and with a 12 hour day/night cycle [19]. Mosquito infections with blood from consenting *P*. *vivax* malaria patients were conducted in Manaus, a Brazilian endemic area for malaria, as described in [21], in accordance with relevant guidelines and regulations. Informed consent was obtained from all subjects. Briefly, *P*. *vivax-* malaria patients were diagnosed by microscopic examination of Giemsa-stained blood smears. The mosquitoes were artificially fed on infected or control blood at 37°C constant temperature, maintained using a water circulation system, to prevent exflagellation of microgametocytes. After the artificial feeding, the infected and non-infected mosquitoes were kept in cages and given 10% sucrose *ad libitum*. All experimental protocols were approved by the Brazilian Ministry of Health, National Council of Health, and National Committee of Ethics in Research (CONEP), written approval number 3726.

To infect mosquitoes with the Gram-positive *Staphylococcus aureus* or Gram-negative *Enterobacter asburiae*, one OD 600 of bacterial culture growth overnight (approximately 10^9^ bacteria ml^-1^) was washed twice in phosphate-buffered saline (PBS) and resuspended in 1 ml of bicarbonate-buffered saline-agarose (BBSA) [22].

### RACE and searching for GATA sequences in the *A*. *aquasalis* partial genome

To obtain the 5’ and 3’ ends of the GATA cDNA, the SMART cDNA RACE (Becton Dickinson Clontech) amplification technique was used. All amplicons generated by the RACE PCR reaction were cloned into the pGEM-T Easy Vector (Promega) and used to transform competent *Escherichia coli* DH5α. Plasmids were sequenced in an ABI 3700 sequencer (Applied Biosystems) at the PDTIS/FIOCRUZ Sequencing Platform. The sequences obtained were used to mount the cDNAs of *A*. *aquasalis* GATA with the CAP3 Sequence Assembly (http://pbil.univ-lyon1.fr/cap3.php ) and Clustal W Programs (http://www.ebi.ac.uk/Tools/clustalw2/). This first GATA sequence was used to search for GATA sequences in the *A*. *aquasalis* genome. This Whole Genome Shotgun project was deposited at DDBJ/ENA/GenBank under the accession number NJHH00000000. Version NJHH01000000 was used in this paper.

The *A*. *gambiae* (Pest strain) and *D*. *melanogaster* genomes and annotated genes were downloaded from NCBI. All sequences were formatted for a Stand Alone Blast [23] running in a Dell server (8 cores; 32GB Ram). To annotate the GATA genes, a reciprocal BLAST approach was used. Firstly, to find the scaffold containing the GATA gene, the *A*. *aquasalis* GATA cDNA was used in a BLASTn search against *A*. *aquasalis* genome (e-value 0.0; identities 100%). Thereafter BLASTx or tBLASTx searches against *A*. *gambiae* and *D*. *melanogaster* scaffolds and proteins (e-value 0.0001; identities > 50%) were performed. Sequences returned by the BLAST search were then used in a tBLASTn or tBLASTx search against *A*. *aquasalis* scaffolds in order to find other GATA homologs in the genome. *A*. *aquasalis* obtained sequences were then annotated with GeneWise 2 (http://www.ebi.ac.uk/Tools/psa/genewise/), using the closest *A*. *gambiae* homolog as template (parameters Global Mode: ON; Splice Site: Modelled).

To evaluate orthology and paralogy relationships among sequences, a phylogenetic and molecular analysis with all protein sequences obtained in BLAST searches from *A*. *aquasalis*, *A*. *gambiae* and *D*. *melanogaster* was conducted using MEGA 7.0 [24]. Sequences were aligned using “MUSCLE” (Multiple Sequence Alignment) and phylogeny was constructed by Neighbour Joining method (2.000 replicates with complete deletion) [25]. The accession numbers of all sequences used in the phylogenetic analysis can be found in Table 1.

**Table 1:** Accession numbers of amino acid sequences used for phylogeny analysis of the GATA family.

Name in Fig 1	Species	GATA Family	Accession Number
Aaeg GATA Grain	*Aedes aegypti*	Grain	XP_001658239.1
Aaeg GATA Pannier		Pannier	AAW31748.1
Aaqu GATA Serpent (Scaffold1498)	*Anopheles aquasalis*	Serpent	KY614523
Aaqu GATA Pannier (Scaffold1547)		Pannier	KY614521
Aaqu GATA Grain (Scaffold396)		Grain	KY614522
Agam GATA Grain	*Anopheles gambiae*	Grain	AGAP004228-PA
Agam GATA Pannier		Pannier	AGAP002235
Agam GATA Serpent		Serpent	AGAP002238-PB
Amel GATA Grain	*Apis mellifera*	Grain	XP_016769155.1
Amel GATA Pannier		Pannier	XP_001121210.2
Amel GATA Serpent		Serpent	XP_016769882.1
Dmel GATA Grain	*Drosophila melanogaster*	Grain	NP_001262366.1
Dmel GATA Pannier		Pannier	NP_001262620.1
Dmel GATA Serpent		Serpent	NP_001247128.1
Isca GATA Grain	*Ixodes scapularis*	Serpent	XP_002434087.1
Nvec GATA	*Nematostela vectenis*	-	AAR24452.1
Tcas GATA Grain	*Tribolium castaneum*	Grain	NP_001158260.1
Tcas GATA Pannier		Pannier	XP_008200488.1
Tcas GATA Serpent		Serpent	XP_008200495.1

### Semi-quantitative RT-PCR

Total RNA was extracted from *A*. *aquasalis* females either sugar-fed or one to five days after dsRNA injections. Up to 5 μg of RNA were treated with RQ1 RNAse-free DNAse (Promega) and used for first strand cDNA synthesis. The same reaction conditions and primers (GATA and housekeeping ribosomal protein gene 49—RP49) used for RT-qPCR were used here and experiments were performed in biological and technical triplicates. The PCR amplicons were separated in a 2.5% ethidium bromide-containing agarose gel. The intensity of amplified products was measured using ImageJ 1.34s software (http://rsb.info.nih.gov/ij) and plotted for semi-quantitative analysis.

### Real Time PCR (RT-qPCR)

Total RNA of whole mosquitoes from the experimental group (sugar, blood and *P*. *vivax-*infected blood, BBSA, *S*. *aureaus* or *E*. *asburiae*-BBSA, dsß-gal or dsSrp-injected) was extracted and treated with RQ1 RNAse-free DNAse (Promega). The synthesis of cDNA was performed with OligodT primer and SuperScript II Reverse Transcriptase (Invitrogen). When the samples were used to quantify the microbiota, random primers were used instead of OligodT primer. All RTPCR reactions were conducted using the SyberGreen fluorescent probe in an ABI 7000 machine (Applied Biosystems). The PCR cycles used were 50°C 2 min, 95^o^ C 10 min, 95^o^ C 15 sec and 63^o^ C 1 min for 35 times for all reactions. To amplify the GATA-Serpent gene the following primers were used: GATAFwd 5’-ATCTGCTACACGCAGCAGGTGCCAT-3’ and GATARev 5’-TGACGATAGCCCCACTGGAGGGAGT-3’. To amplify the 16S gene, primers described in Lane [26] were used: 16S-F TCC TAC GGG AGG CAG T and 16S-R GGA GTA CCA GGG TAT CTA ATC CTG T. The relative expression of studied genes was determined based on gene expression CT difference formula [27]. Quantifications were normalized in relationship to the housekeeping gene rp49 [28]. All the experiments were performed with four to six biological replicates and three experimental replicates. The statistics method used in the analyses was ANOVA test with multiple comparisons of Tukey or Games-Howell. When the parametric model (ANOVA) was not adequate, we utilized the Mann-Whitney test. All tests were performed with reliability level of 95% (α = 0.05). The statistical analyses were conducted using the GraphPad Prism 5, R, software.

### Gene silencing

GATA-Serpent gene knockdown was performed by the introduction of double stranded RNA (dsRNA) into *A*. *aquasalis* females according to Bahia *et al*. [3]. The dsRNAs were produced with the T7 Megascript kit (Ambion) using RT-PCR-amplified fragments. Amplicons for dsß-gal were produced via plasmid templates and for dsSrp by RT-PCR products, from sugar-fed female cDNA, resulting in 544 bp and 404 bp fragments, respectively. The PCR reactions were performed in two PCR rounds as described [3]. The primers used for the first round of PCR were: ß-galdsRNAFwd 5’- tggcgcccctagatgTGATGGCACCCTGATTGA-3’ and ß-galdsRNARev 5’- tggcgcccctagatgTCATTGCCCAGAGACCAGA-3’, GATAdsRNA Fwd 5’- tggcgcccctagatgACGAGAGTGCGTCAATTGTG-3’ and GATAdsRNA Rev 5’- tggcgcccctagatgGATTGTTTCCATCGTTGGCT-3’. The second round PCR primer, used was 5’-CCGTAATACGACTCACTATAGGtggcgcccctagatg-3’.

Sixty-nine nanoliters of either dsSrp or dsß-gal (3 μg/μl) were intrathoracically injected into cold anesthetized 3–4 day old female mosquitoes by a nano-injector (Nanoject, Drummond) with glass capillary needles. All injected insects were maintained inside a BOD incubator and fed on sugar solution as previously described [3].

### *A*. *aquasalis* survival assays after dsRNA injection

Groups containing at least 40 3-4-day-old *A*. *aquasalis* female mosquitoes injected with either dsß-gal (control) or dsSrp were kept in cages at 27°C with 70% humidity and monitored daily for survival. The day after injection dead mosquitoes were removed from the cages. Survival percentages represent the mean of three biological replicates. Statistical significances were determined by Kaplan-Meier survival analysis using GraphPad Prism 5 software (GraphPad, La Jolla, California, USA) and p-values were determined by the Wilcoxon test.

### Quantification of the midgut native microbiota

Prior to dissection the mosquitoes were sterilized by immersion in 70% ethanol for 2 min and subsequently rinsed five times for 1 min in phosphate-buffered saline. Five to six midguts were dissected with aseptic forceps over sterile glass slides in sterile PBS four days after dsRNA injection, grinded and seeded on LB agar plates after serial dilutions.

Two days after incubation at room temperature the total number of bacteria per plate was counted and used to determine the number of colony forming units (CFU). Three independent experiments were performed. To quantify the total microbiota (cultivable and non-cultivable), we amplified the 16S rRNA gene of the total bacteria gut from 4-days dsSrp and dsß-gal (control) injected mosquitoes using RT-qPCR as described above.

### *Plasmodium* quantification after dsRNA injection

Two to three days after the dsRNA injections, depending on patient availability, the mosquitoes were fed with *P*. *vivax* infected blood. Three to five days post infection (dpi) the midguts were dissected and stained with 2% merbromin, and the number of oocysts determined under a light microscope. At least 30 midguts were used for each experimental condition. Oocyst numbers in dsSrp-injected insects were compared to the control ones, injected with dsß-gal. The results of three independent experiments were pooled together. The significance of gene silencing on oocysts loads was determined by the Mann-Whitney statistical test. The Chi-Square test was used to determine difference in the infection prevalence.

### Hemocyte collection, morphological identification and quantification

To evaluate any effects in the differentiation and proliferation of the hemocytes, sugar-fed adult female *A*. *aquasalis* were injected with dsRNA (Srp or β-gal) and three days later hemocytes were collected through hemolymph perfusion. For such, mosquitoes were intrathoracically injected with an anticoagulant solution containing 60% Schneider’s Insect medium, 30% citrate buffer and 10% fetal bovine serum, and 10μl were then recovered with a sterile pipette through an incision made between the last 2 segments of the mosquito abdomen [13]. The collected sample was deposited in a sterile disposable hemocytometer slide (10μl capacity, Neubauer Improved, iNCYTO C-Chip DHC-N01) and the hemocytes were photographed, identified and counted using a light microscope (40x objective) by morphological identification and counting of the three distinct cell types found in the hemolymph samples (prohemocytes, oenocytoids, and granulocytes). Total number and proportion of cells were determined for each individual mosquito. The statistical analyses were performed using GraphPad Prism 5 (GraphPad, La Jolla, California, USA) from three independent biological replicates. Hemocyte numbers and proportions analyses were performed using Student’s t-test and significance was assessed at p<0.05.

## Results

### Searching for *A*. *aquasalis* GATA TFs

Three GATA sequences (accession numbers GR486699, GR486641, GR486542) were identified by subtraction between two libraries: *A*. *aquasalis* 2 hours post feeding on uninfected and *P*. *vivax* infected blood [22]. One single sequence of 174 bp was obtained after the alignment of those three sequences. The SMART cDNA RACE amplification technique was used to obtain a sequence of 725 bp for the *A*. *aquasalis* GATA gene, which included an open reading frame encoding a 209 amino acid residues protein plus a 124bp upstream untranslated region. The GATA sequence was located in scaffold 1498 of the *A*. *aquasalis* genome. Using *A*. *gambiae* GATA sequences as queries (AGAP002238-PB, AGAP004228-PA and AGAP002235-PA) by reciprocal BLAST, two other GATA genes were found in scaffolds 1547 and 396 of the *A*. *aquasalis* genome.

**Fig 1:**
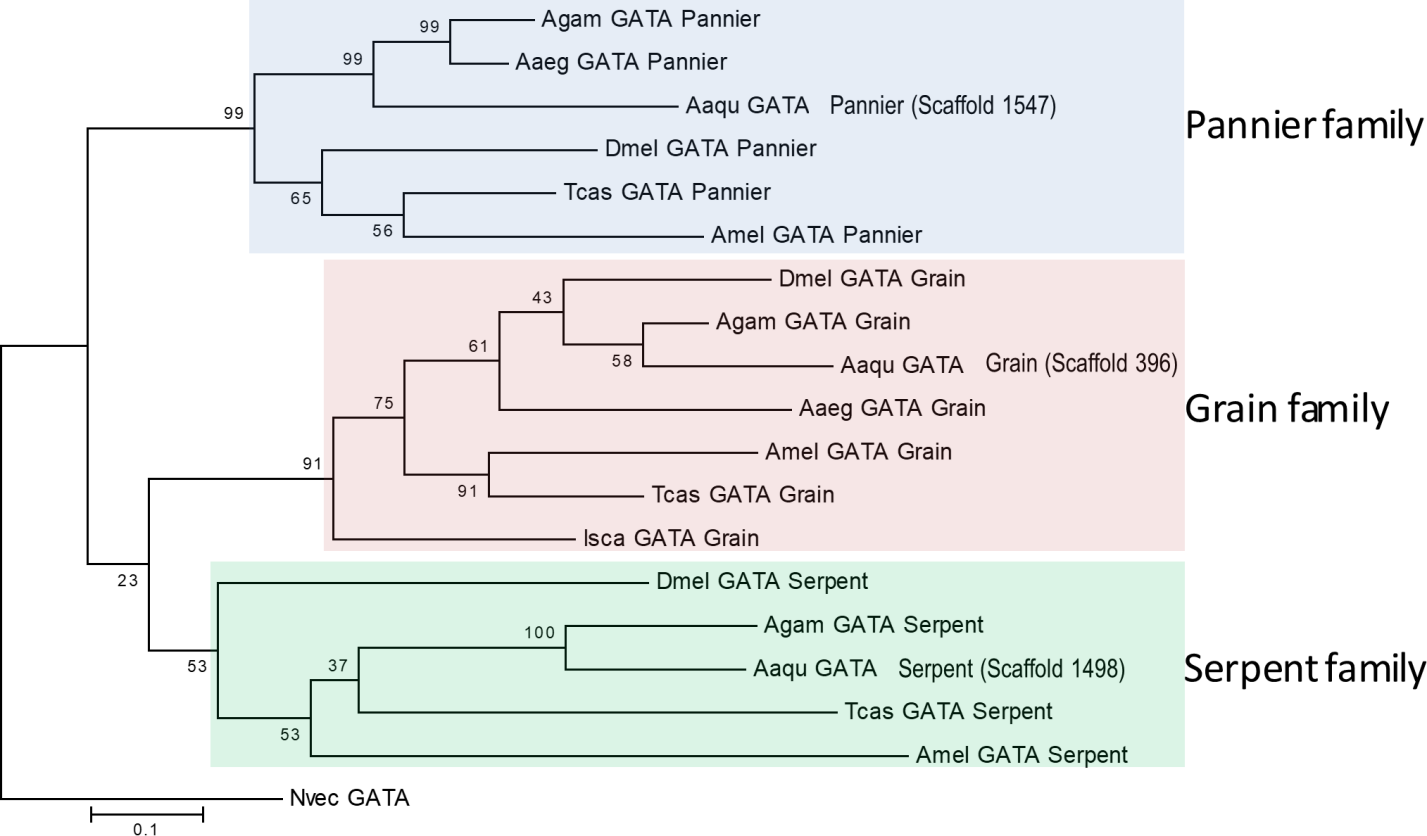
Characterization of *A*. *aquasalis* GATA transcription factors. The evolutionary history of GATA genes was inferred using the Neighbor-Joining method. The red arrow points to *AaquGATA* gene (Scaffold1498). Colored boxes highlight sequences of the three GATA families (Pannier, Grain and Serpent). The percentage of replicate trees in which the associated taxa clustered together in the bootstrap test (2000 replicates) is shown next to the branches. The analysis involved 13 amino acid sequences. All positions containing gaps and missing data were eliminated. There was a total of 139 positions in the final dataset. The accession number for each sequence is included in S1 Table.

**Fig 2:**
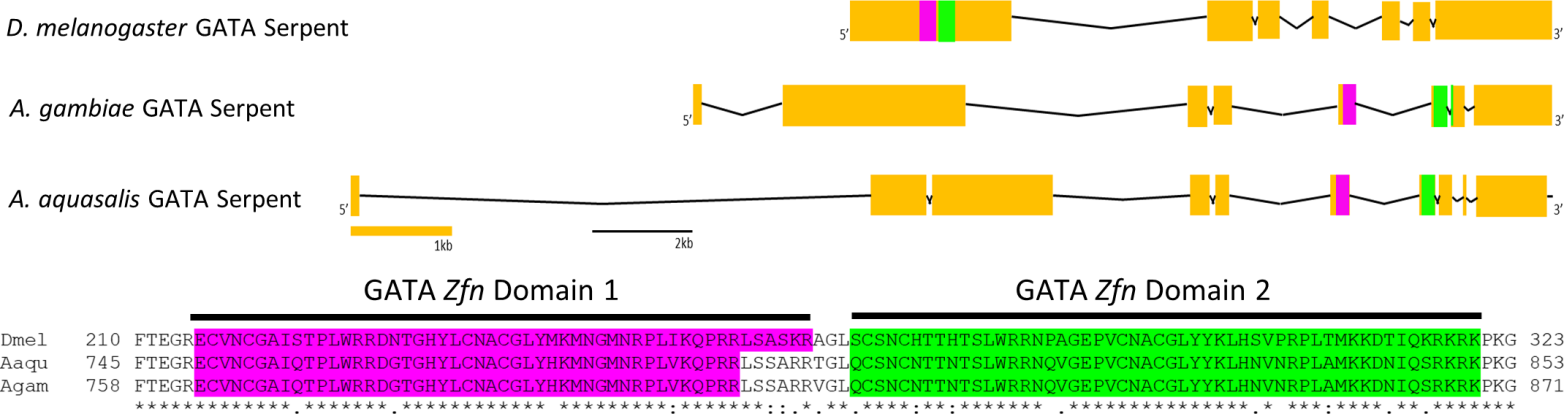
Representation of *A*. *aquasalis* GATA Serpent structure. The Fig shows the representation of GATA-Serpent transcripts of *D*. *melanogaster*, *A*. *gambiae* and *A*. *aquasalis*. Yellow boxes represent exons and lines represent introns. Both zinc fingers domains are highlighted in pink (first domain) and green (second domain). The underlined amino acids alignment shows the conservation of the two zinc finger DNA binding domains.

Phylogenetic analyses suggest that the previously identified gene [22] is a GATA Serpent gene orthologous to *A*. *gambiae* (AGAP002238) and *D*. *melanogaster* (NP_001262366.1) genes (Figs 1 and 2); while the genes identified with bioinformatic tools are orthologous to GATA Pannier and Grain (AGAP002235 and NP_001247128.1; and AGAP004228 and NP_001262620.1, respectively) (Fig 1).

The three *A*. *aquasalis* sequences contain two zinc finger binding domains characteristic of the GATA superfamily. These domains are shown for the GATA- Serpent gene in Fig 2. The *A*. *aquasalis* GATA genes have been deposited in the NCBI database under accession numbers KY614521, KY614522 and KY614523.

Using the *A*. *gambiae* AGAP002238 amino acid sequence as template, we performed a BLAST search for homologous sequences in the *A*. *aquasalis* genome. Coding DNA sequences (CDS) for *A*. *aquasalis* gene were annotated with GeneWise. We obtained a complete 3,417 nucleotides AaquGATA-Serpent CDS generating a 1,139 amino acids sequence (Fig 2). Thereafter this gene was named as *Aaqu-Srp*.

### *Aaqu-Srp* transcription is induced after *P*. *vivax* but not after oral bacterial infection

**Fig 3:**
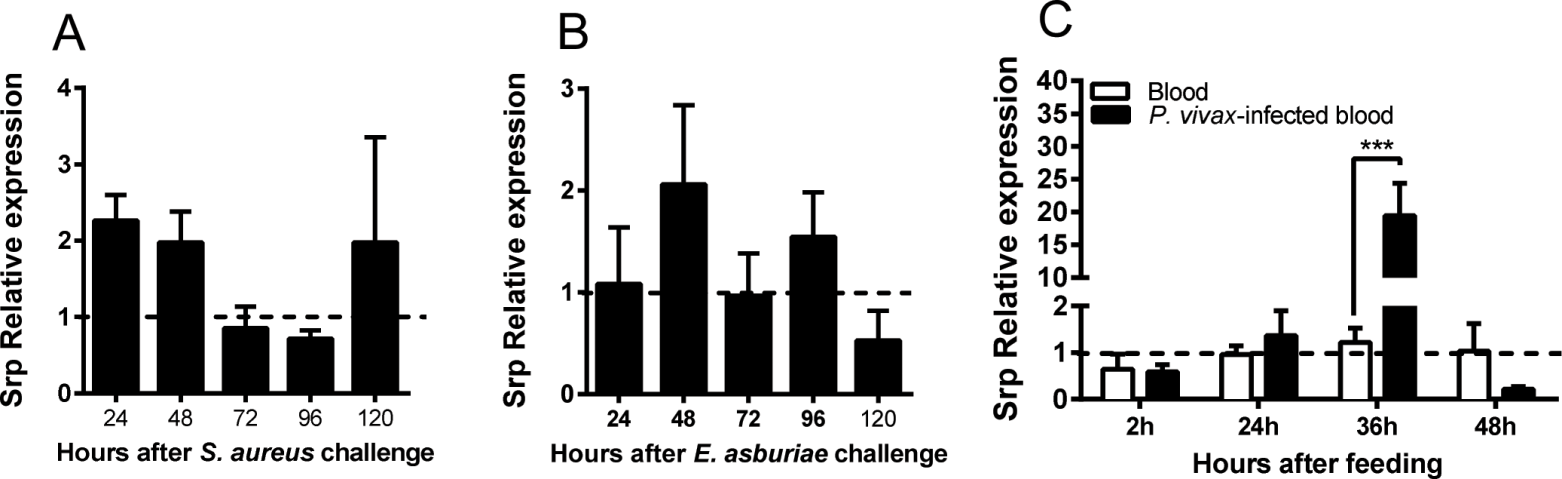
Validation of *A*. *aquasalis Aaqu-srp*. Expression levels of GATA-Serpent transcription factor in *A*. *aquasalis* whole body following different feeding regimens. mRNA expression of GATA-Serpent in females 1–5 days after *S*. *aureaus* (A) and *E*. *asburiae* (B) challenges; and (C) blood-fed females and *P*. *vivax* infection. The dotted line represents the expression of GATA-Serpent in sugar-fed mosquitoes. h: hours. +–: s.e.m.; * p < 0.05, ** 0.03 < p < 0.01, *** p < 0.01. Six samples were used originating from three biological replicates.

To understand the role of *Aaqu-Srp* in the *A*. *aquasalis* immune response, we studied the expression of the gene in adult female mosquitoes after feeding on sugar, blood from healthy volunteers and from *P*. *vivax*-infected patients, and BBSA and BBSA containing Gram-positive (*S*. *aureaus*) or Gram-negative (*E*. *asburiae*) bacteria. RT-qPCR results showed that *Aaqu-Srp* is not significantly induced after bacterial infection (Fig 3A and 3B), but it is highly induced (almost 15 times) 36 h after *P*. *vivax* infection, in contrast with all other time points analyzed (sugar-fed females, 2 and 24 h after blood-feeding and infection, 36 h after blood-feeding and bacterial infections) (Fig 3A–3C).

### *Aaqu-Srp* affects *A*. *aquasalis* survival and is involved in anti-bacterial defense

**Fig 4:**
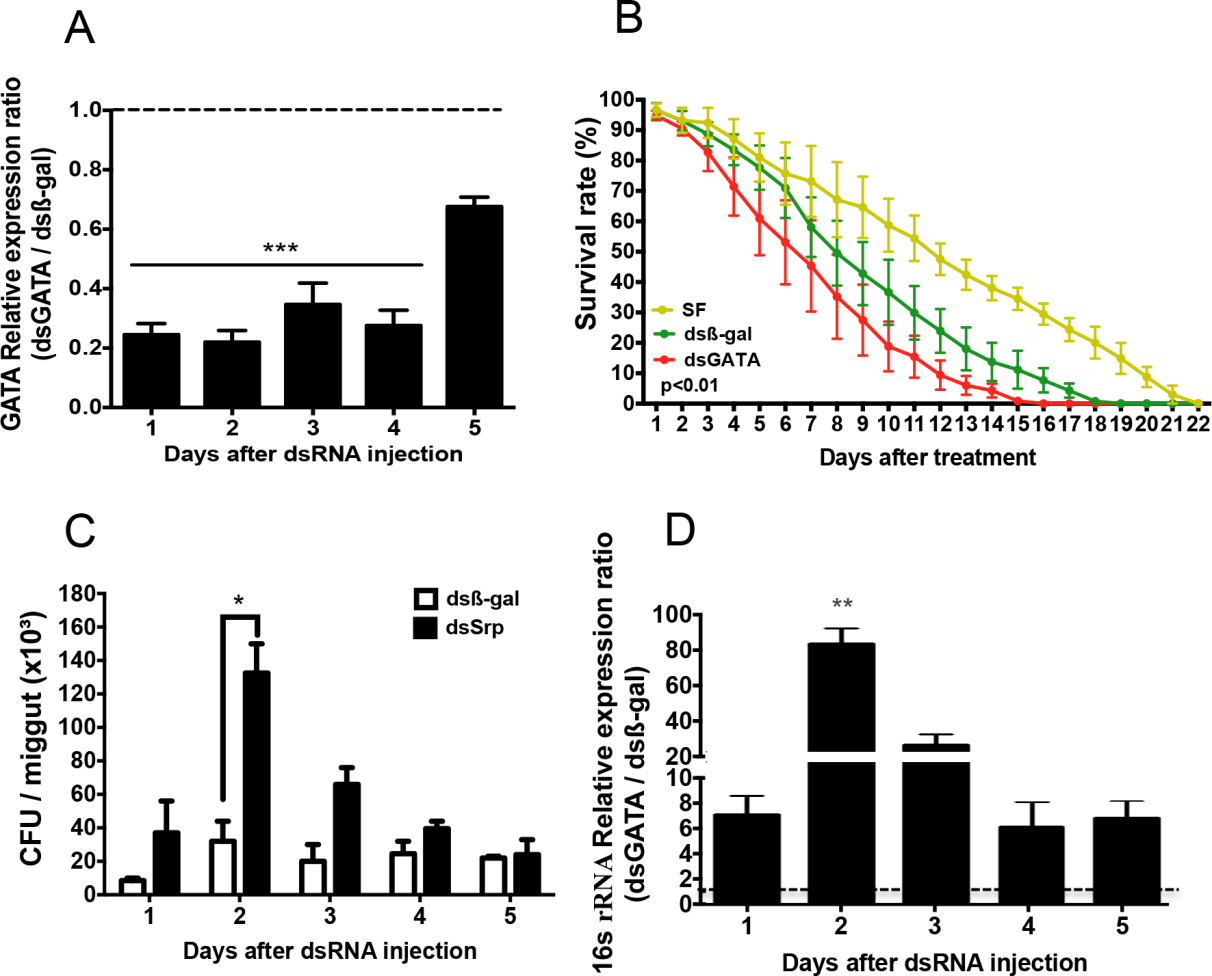
GATA-Serpent gene silencing in *A*. *aquasalis*. (A) Effect of dsSrp injections on GATA-Serpent and on the housekeeping RP49 gene mRNA expression. (B) Life span of mosquitoes 3–22 days after injections with dsSrp and dsβ-gal (control) maintained on 10% sucrose solution. The survival rates were analyzed by Kaplan-Meier survival analysis with Wilcoxon test to determine the significance. Detailed statistical information is shown in S1 Table. Three independent experiments were performed with at least 35 mosquitoes in each group. (C-D) Evaluation of cultivable bacterial population in mosquitoes injected with dsβ-gal and dsSrp. Relative quantification of 16S rRNA of culture-independent microbiota (C) and colony forming units of cultivable bacteria (D) in mosquitoes injected with dsSrp/dsβ-gal. More than ten insects from three independent experiments were evaluated in C and D. 1-5d: females 1 to 5 days after dsRNA injection.

Gene silencing was performed by injecting dsRNA for *β-galactosidase* (β-gal), a gene not present in the mosquito genome, and *Aaqu-Srp* in *A*. *aquasalis* females. Semi-quantitative RT-PCR and RT-qPCR showed a 70–85% decrease of *Aaqu-Srp* mRNA levels during the first four days after dsRNA injection and a return to normal 5 days after inoculation (Figs 4A and S1). After each challenge, mosquitoes were monitored daily for survival. We observed an average decrease of 3 days in mosquito survival after injection of dsSrp compared with dsβ-gal (control) (Figs 4B and S2, S1 Table). To assess the role of *Aaqu-Srp* as an immune inducer and provide the possible explanation of the increased mosquito mortality when *Aaqu-Srp* is depleted, we have evaluated the proliferation of mosquito natural midgut microbiota when *Aaqu-Srp* gene was silenced. To that end we plated and evaluated 16s rRNA expression in injected mosquitoes midgut 1–5 days after dsSrp and dsβ-gal injection. We observed a significant increase of bacteria in mosquito midguts 48 h after dsSrp injection (Figs 4C, 4D and S3).

### *Aaqu-Srp* is determinant of resistance to *P*. *vivax* infection

**Fig 5:**
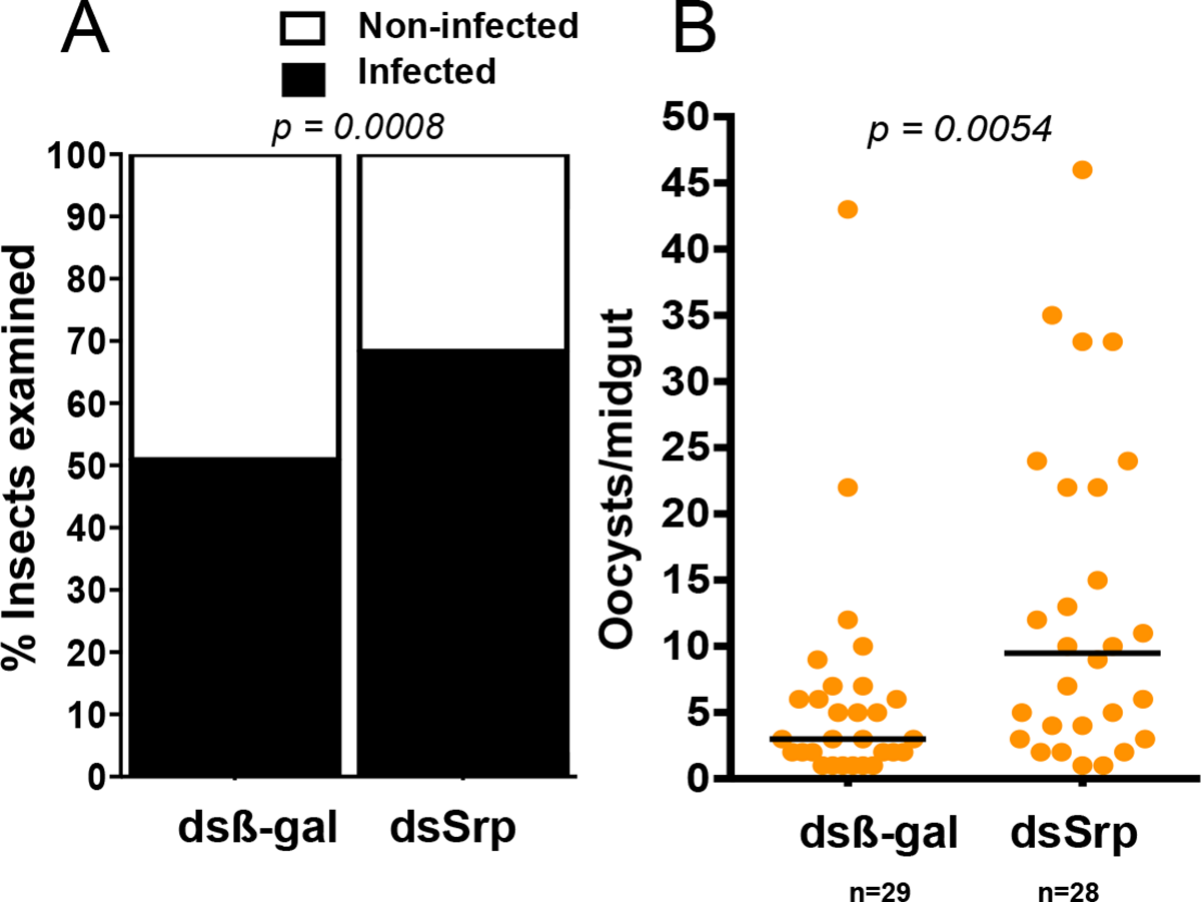
*Plasmodium* infection of GATA-Serpent-deficient mosquitoes. (A) Percentage of infected mosquitoes after β-gal and GATA-Serpent dsRNA injection. The Chi-Square (and Fisher’s exact) test of was used to determine the significance. (B) *P*. *vivax* oocyst numbers in mosquito midguts that received dsβ-gal and dsSrp injections 3–5 days after *Plasmodium* infection. The significance of gene silencing effect on oocyst loads in experimental samples, compared to dsβ-gal -treated control, was determined by Mann-Whitney test (Supporting Information S3 Table). Horizontal red lines indicate median infection intensity and orange dot represent each specimen analyzed. Three independent experiments were performed. Here only *P*. *vivax* positive samples are represented.

RNAi-mediated *Aaqu-Srp* depletion in mosquitoes experimentally fed on patient blood containing *P*. *vivax* parasites resulted on an increase in the prevalence of *P*. *vivax*-infected mosquitoes after Serpent knock-down from 51% insects in the control group to 68% in the dsSrp injected mosquitoes (Fig 5A). A significantly increased *P*. *vivax* load was also observed (Figs 5C and S4). The median oocyst number per mosquito midgut increased from three in the control (β-gal) to 9.5 in the experimental (Srp) group (Fig 5C and S3 Table).

### *Aaqu-Srp* is involved in hemocyte differentiation

**Fig 6:**
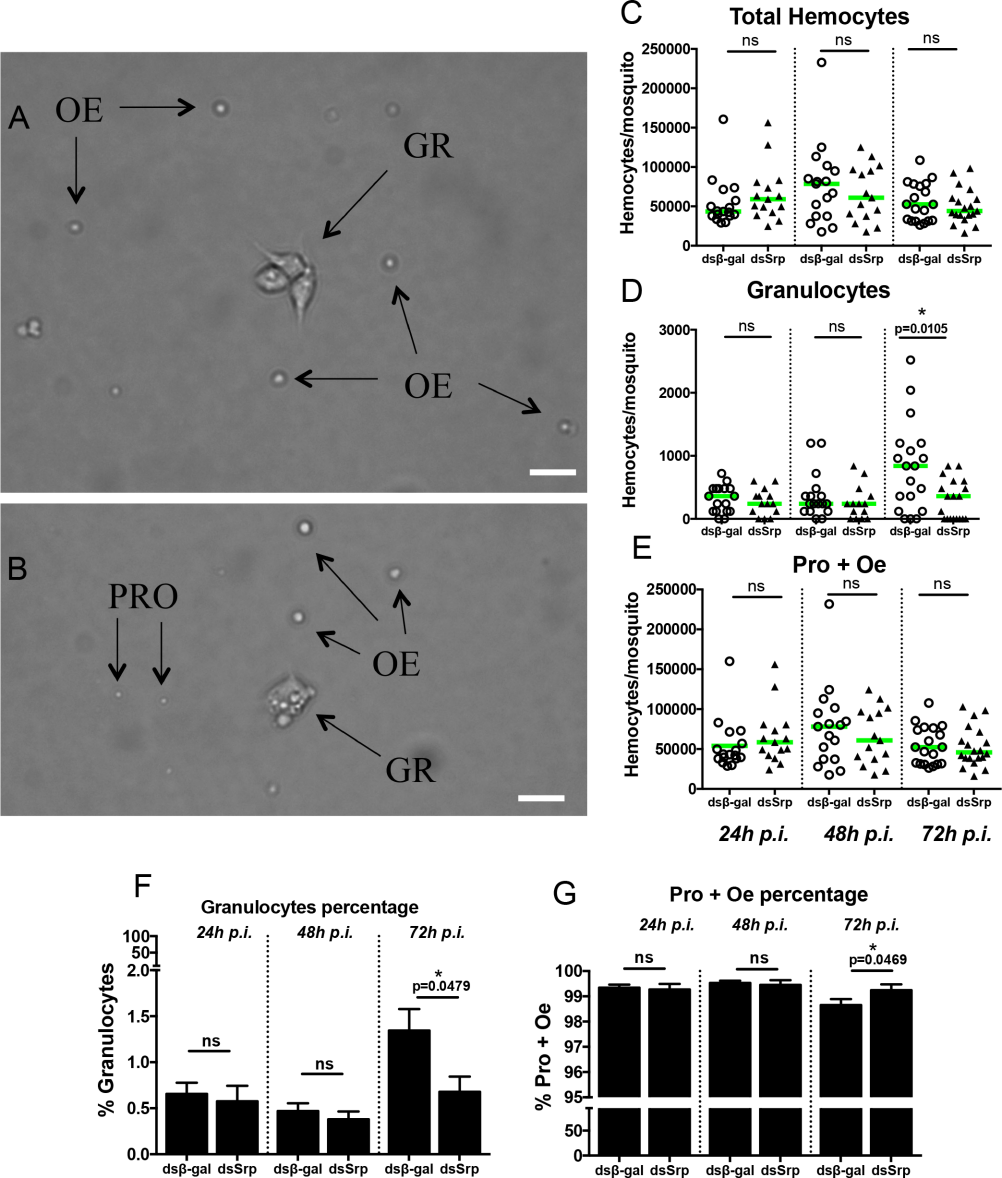
Effect of *A*. *aquasalis* GATA-Serpent gene in differentiation of hemocytes. (A-B) representative light microscopy images of hemocyte types from *A*. *aquasalis*, prohemocytes (PRO), granulocytes (GR), oenocytoids (OE). (C-E) Number of total hemocytes (C), granulocytes (D), and prohemocytes and oenocytoids (E), and percentage of granulocytes (F) or prohemocytes and oenocytoids (G) obtained from mosquitoes injected with dsSrp or dsβ-gal (control) at different timepoints (24, 48 and 72 h) post dsRNA injection (p.i.). Hemocyte numbers are plotted as the median and each individual mosquito is represented as a dot (∼ 20 mosquitoes for each experimental condition). The data in the Fig represents the combination of three independent experiments. Statistical analysis was performed using the Mann–Whitney test to determine significance. ns, not significant. * p < 0.05.

In order to study the role of *Aaqu-Srp* in *A*. *aquasalis* immune response against *P*. *vivax* and bacteria, we injected mosquitoes with dsRNA for β-gal or *Aaqu****-****Srp* genes and, after 3 days, we counted hemocyte numbers. Since there are no previously reported studies describing *A*. *aquasalis* hemocytes, we used optical light microscopy to identify the different morphotypes. *A*. *aquasalis* has three types of circulating hemocytes in the hemolymph that differ with respect to morphology and size (Fig 6A and 6B). The prohemocytes and oenocytoids have circular shapes, differing only in size and light refraction when observed by light microscopy (Fig 6A and 6B). The prohemocytes are smaller compared with oenocytoids and more refractory (Fig 6A and 6B). Granulocytes are polymorphic and adhere quickly to the glass slide. Many granules were seen in the cytoplasm of these cells (Fig 6A and 6B).

After establishing methods to identify the *A*. *aquasalis* hemocytes cell types, we tested the effect of *Aaqu-Srp* silencing on cellular immune response. For that, we quantified the *A*. *aquasalis* hemocyte cell types from sugar-fed insects three days after injection with dsRNA. The results showed a decrease in granulocytes numbers in dsSrp injected mosquitoes only 72 hours post dsRNA injection (Fig 6D and 6F). Total number of hemocytes and prohemocytes and oenocytoids did not show any difference between the experimental groups in any timepoint analyzed (Fig 6C and 6E).

## Discussion

GATA is a family of transcription factors important in development and differentiation of a variety of organisms that go from mammals to plants. In humans, flies and worms these proteins are also important in immunity [8–10, 29]. Here we investigated whether a GATA-Serpent family gene (*Aaqu-Srp*) plays a role in *A*. *aquasalis* immunity against bacteria and *Plasmodium*. In the *A*. *aquasalis* partial genome three different GATA genes (Serpent, Pannier and Grain) were found, as opposed to the five genes observed in *D*. *melanogaster* and *A*. *gambiae*. It appears that *A*. *aquasalis* has lost two GATA TFs of the 456 cluster, the DmelGATAe or and Agam456a and DmelGATAd or and Agam456bbb orthologs. Nevertheless, the finalization of the *A*. *aquasalis* genome sequencing and annotation will bring a better light on this subject.

The expression of *Aaqu-Srp* did not suffer any significant alteration after ingestion of blood and bacterial infection, although mRNA levels were highly increased after infection by *P*. *vivax*. Differently from other *Anopheles–Plasmodium* models [e. g. ref. 30], we observed in previous studies that the passage of *P*. *vivax* through the *A*. *aquasalis* midgut did not trigger a strong immune response, but the exposure of the parasite to the hemolymph was responsible for the immune activation [3, 22, 31]. Thus the induction of GATA-Serpent 36h post infection could be a response to the presence of early oocysts in the midgut basal lamina, facing the hemolymph, rather than the midgut invasion that would have happened between 18–24 h post feeding.

To confirm the immune role of Aaqu-Srp, reverse genetic experiments to knock down the expression of the *Aaqu-Srp* gene in *A*. *aquasalis* were successfully performed. An increased mortality was detected in mosquitoes injected with dsSrp compared with dsβ-gal control. The *Aaqu-Srp* knocked-down mosquitoes died earlier than the control group, potentially due to an increase of the bacterial load in the mosquito midgut as observed by both LB-Agar culture and 16S rRNA gene amplification. These results showed the involvement of the *Aaqu-Srp* in *A*. *aquasalis* anti-bacterial immune response although we did not observe a significant increase of *Aaqu-Srp* transcript levels in mosquitoes infected with either Gram-positive or negative bacteria. The possible explanation for this finding is the specific challenge of the mosquito with Gram-negative or positive bacteria in the RT-qPCR experiments as compared with the variety of bacteria normally found in microbiota present in mosquitoes used in the RNAi experiment. It appears that this gene is important for the insect to sense and control the associated microbiota.

We tested the effect of *Aaqu-Srp* silencing on *Plasmodium* infection. The number of infected mosquitoes and the median of *P*. *vivax* oocysts per individual increased significantly after *Aaqu-Srp* gene silencing, indicating that this gene is also involved in anti-*Plasmodium* responses. These results provide evidence that *Aaqu-Srp* is important for protecting *A*. *aquasalis* against bacterial and *P*. *vivax* infection. *D*. *melanogaster* GATA-Serpent functions as the major GATA TF in immature stages and adult flies fat body, and is essential for immune response activation in this tissue [8–10]. As with *D*. *melanogaster* Serpent, we expect that the *Aaqu-Srp* may induce some effector genes in the fat body contributing to mount a robust immune response against *P*. *vivax* and bacterial infections.

In order to scrutinize the mechanisms by which *Aaqu-Srp* is capable of protecting *A*. *aquasalis* mosquito against *Plasmodium* infections, we tested the effect of *Aaqu-Srp* silencing on the mosquito cellular immune responses by hemocyte quantification. We identified three cellular types circulating in the *A*. *aquasalis* hemolymph: prohemocytes, oenocytoids and granulocytes similar to cells previously described in other mosquito species [13, 14, 32, 33]. Based on functional bioassays and markers granulocytes were described as cells with numerous granules in the cytoplasm, with phagocytic properties and capable of binding to foreign surfaces [13, 32]. *A*. *aquasalis* granulocytes showed polymorphic, contained several granules in the cytoplasm and adhered to glass slides. Oenocytoids were described in *A*. *gambiae* and *A*. *aegypti* as having a rounded shape, a homogeneous cytoplasm, as being non-phagocytic and showing phenoloxidase activity [13, 32]. On the other hand, prohemocytes are bigger, round and uniform in size, have a large nuclear to cytoplasmic ratio, and do not present either phagocytic or phenoloxidase activity. Similarly, *A*. *aquasalis* oenocytoids and prohemocytes showed a circular morphology, differing only in size and light refraction under the microscopy.

Hemocyte counting was performed in sugar-fed mosquitoes 24 h, 48 h and 72 h after *Aaqu-Srp* silencing. A significant decrease in granulocyte numbers was observed in the dsSrp treated group of mosquitoes. Although at 48 h we observed a lower number of granulocytes in both dsβ-gal- and dsSrp-injected mosquitoes, up to 72 h post-silencing there was an increase in granulocyte number only in the control group, implicating GATA-Serpent in granulocyte differentiation.

Based on our results we could state that GATA-Serpent silencing in *A*. *aquasalis* had an effect on the insect microbiota, with a peak at 48 h after dsRNA injection. In parallel, GATA-Serpent silencing also resulted in increased *P*.*vivax* oocyst numbers 3–5 days post infection. GATA-Serpent silencing also affected granulocyte numbers, pointing to a possible role of this transcription factor on controlling hemocyte differentiation in our mosquito model. These results suggest that differentiation of *A*. *aquasalis* granulocytes promoted by *Aaqu-Srp* might impact on infection by *P*. *vivax* and microbiota control.

In *Drosophila*, the JAK-STAT pathway has an essential role in hematopoiesis. Minakhina *et al*. [33] showed that hemocytes lacking STAT fail to differentiate into plasmatocytes and that the GATA factor Pannier is a downstream target of STAT. Agaisse *et al*. [34] observed that hemocytes release *upd3*, which activates the JAK-STAT pathway. In *A*. *gambiae* mosquitoes, STAT is involved in prohemocytes differentiation into both oenocytoids and granulocytes and anti-*Plasmodium* defense [18, 35]. We showed previously that JAK-STAT pathway is important in *A*. *aquasalis* immune response against *P*. *vivax* [3]. It is possible that the JAK-STAT pathway is also involved in *P*. *vivax* control by regulating GATA-Serpent transcription factor and hemocyte proliferation and differentiation into granulocytes in *A*. *aquasalis*. Thus, further investigation is necessary to confirm the involvement of JAK-STAT in the *Aaqu-Srp* activation or vice-versa.

In summary, we showed here that Aaqu-Srp presents an immune role in controlling *P*. *vivax* infection and microbiota in the *A*. *aquasalis*, a competent Neotropical malaria vector in nature. We also showed that the *Aaqu-Srp* gene is capable of regulating hemocytes differentiation and may act by amplifying and activating the immune response in the mosquito hemolymph upon infection.

## Supporting information

S1 Fig Effect of dsSrp injections on GATA-Serpent and on the housekeeping RP49 gene mRNA expression on sugar-fed mosquitoes and sugar-fed and injected with dsRNA (1d-5d). M–nolecular marker, SF–sugar-fed, d–days after injection.
(TIF)

S2 Fig Kaplan–Meier survivorship curve comparing insects injected with dsβ-gal and dsSrp. Mosquitoes were raised under similar conditions after dsRNA treatment. Three biological replicates (rep1-3) were done and are shown here. Kaplan–Meier survival analysis was used together with the log-rank test to determine the *P*-values, and *p* < 0.05 indicates significance (Supporting Information S1 Table).
(TIF)

S3 Fig Evaluation of cultivable bacterial population from midgut of mosquitoes injected with dsβ-gal and dsSrp treated or not with antibiotics by counting the colony forming units (CFU) in LB agar plates. Antibiot.–antibiotics.
(PPTX)

S4 Fig *Plasmodium* infection of GATA-Serpent-deficient mosquitoes. (A) Percentage of infected mosquitoes after β-gal and GATA-Serpent dsRNA injection. The Chi-Square (and Fisher’s exact) test of was used to determine the significance. (B) *P*. *vivax* oocyst numbers in mosquito midguts that received dsβ-gal and dsSrp injections 3–5 days after *Plasmodium* infection. The significance of gene silencing effect on oocyst loads in experimental samples, compared to dsβ-gal -treated control, was determined by Mann-Whitney test (Supporting Information S3 Table). Horizontal red lines indicate median infection intensity and orange dot represent each specimen analyzed. Three independent experiments were performed.
(TIF)

S1 Table Accession numbers of amino acid sequences used for phylogeny analysis of the GATA family. (DOCX)

S2 Table Survival analysis of *A*. *aquasalis* mosquitoes injected with dsRNA for *Aaqu-Srp* or β-gal. p*: The blue highlighted wells indicate the significance and survival rates between the *A*. *aquasalis* injected with dsSrp compared to dsβ-gal (control).
(DOCX)

S3 Table Detailed information and statistical analysis of oocysts in *Aaqu-Srp* gene knockdown mosquitoes. P*: The blue highlighted wells indicate the significance of oocysts loads between mosquitoes injected with β-gal dsRNA (control) and *Aaqu-Srp* dsRNA.
(TIF)
