# Supplementary figures and images for: An Anopheles aquasalis GATA factor Serpent is required for immunity against Plasmodium and bacteria
Source: PLoS Negl Trop Dis. 2018 Sep 24;12(9):e0006785. doi: 10.1371/journal.pntd.0006785 (PMC6171954; doi:10.1371/journal.pntd.0006785)

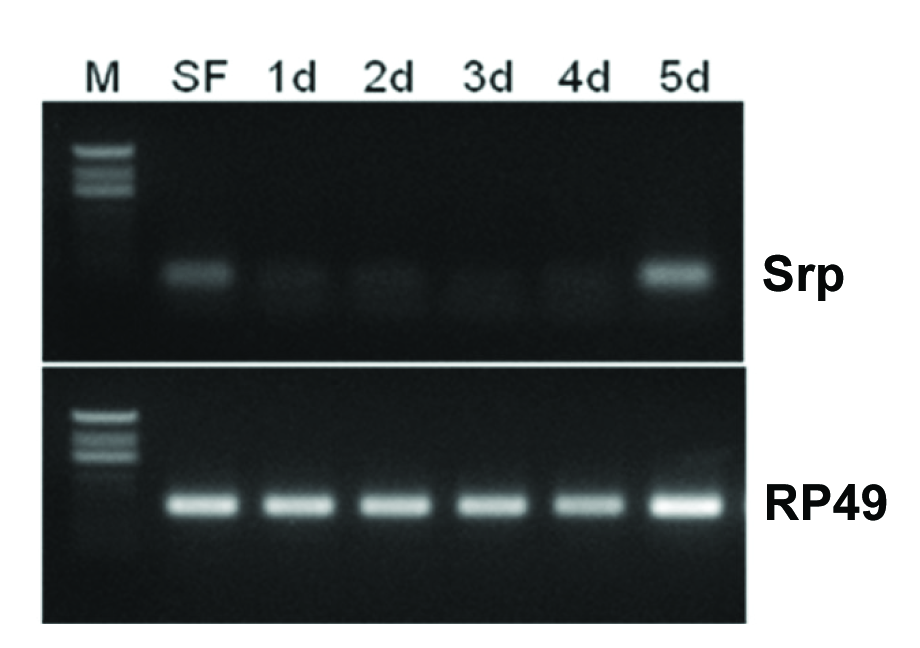

Supplement: S1 Fig — M–nolecular marker, SF–sugar-fed, d–days after injection. (TIF) [file pntd.0006785.s001.tif]

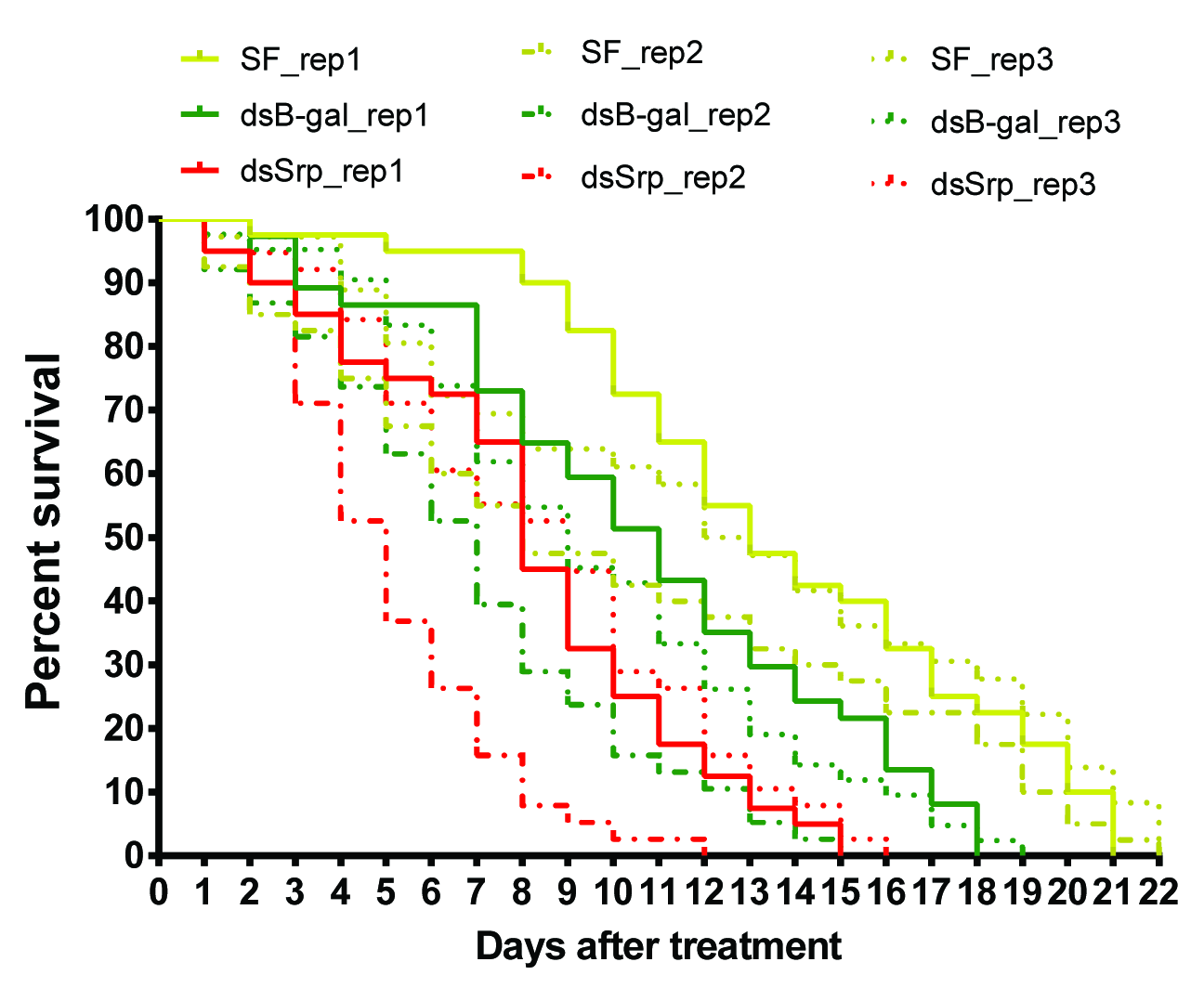

Supplement: S2 Fig — Mosquitoes were raised under similar conditions after dsRNA treatment. Three biological replicates (rep1-3) were done and are shown here. Kaplan–Meier survival analysis was used together with the log-rank test to determine the P-values, and p < 0.05 indicates significance (Supporting Information S1 Table). (TIF) [file pntd.0006785.s002.tif]

## Slide 1
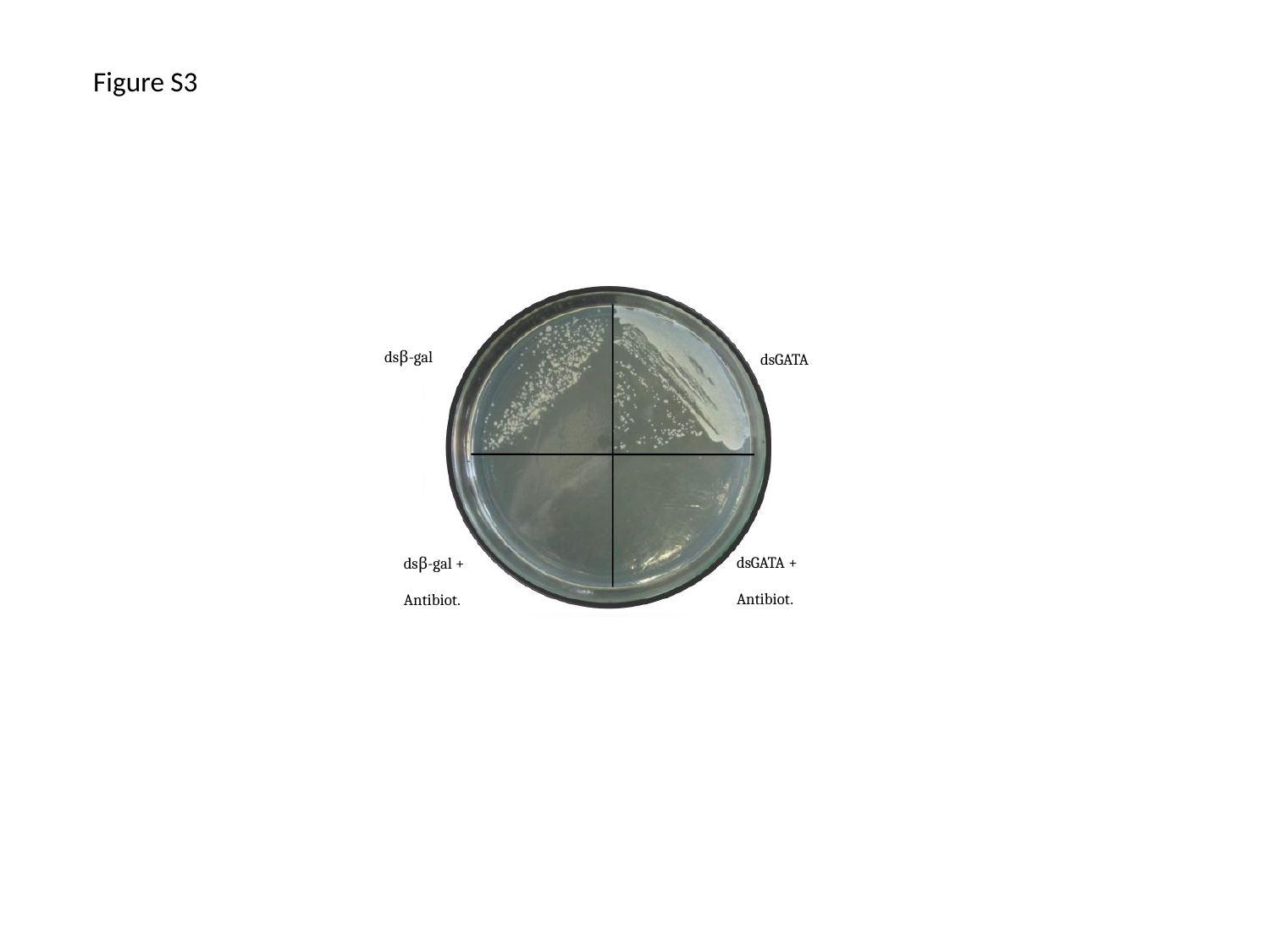

Figure S3
dsβ-gal
dsGATA
dsGATA +
Antibiot.
dsβ-gal +
Antibiot.

Supplement: S3 Fig — Antibiot.–antibiotics. (PPTX) [file pntd.0006785.s003.pptx]

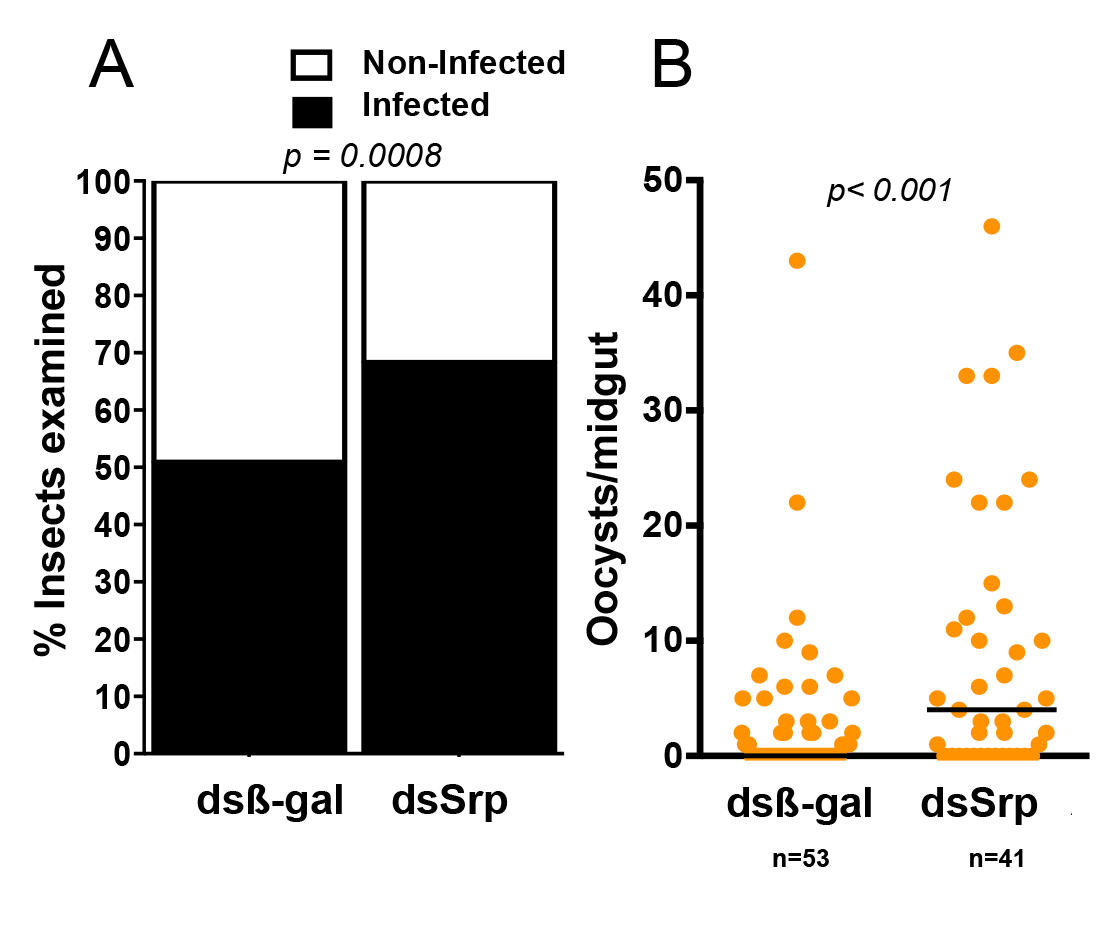

Supplement: S4 Fig — (A) Percentage of infected mosquitoes after β-gal and GATA-Serpent dsRNA injection. The Chi-Square (and Fisher’s exact) test of was used to determine the significance. (B) P. vivax oocyst numbers in mosquito midguts that received dsβ-gal and dsSrp injections 3–5 days after Plasmodium infection. The significance of gene silencing effect on oocyst loads in experimental samples, compared to dsβ-gal -treated control, was determined by Mann-Whitney test (Supporting Information S3 Table). Horizontal red lines indicate median infection intensity and orange dot represent each specimen analyzed. Three independent experiments were performed. (TIF) [file pntd.0006785.s004.tif]

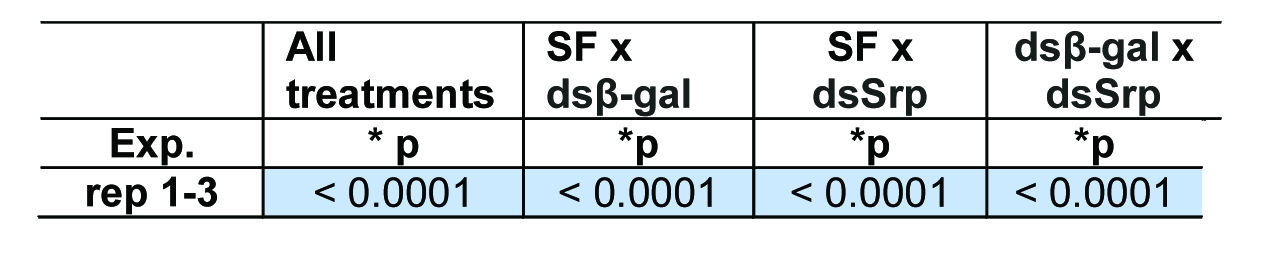

Supplement: S2 Table — p*: The blue highlighted wells indicate the significance and survival rates between the A. aquasalis injected with dsSrp compared to dsβ-gal (control). (DOCX) [file pntd.0006785.s006.docx]

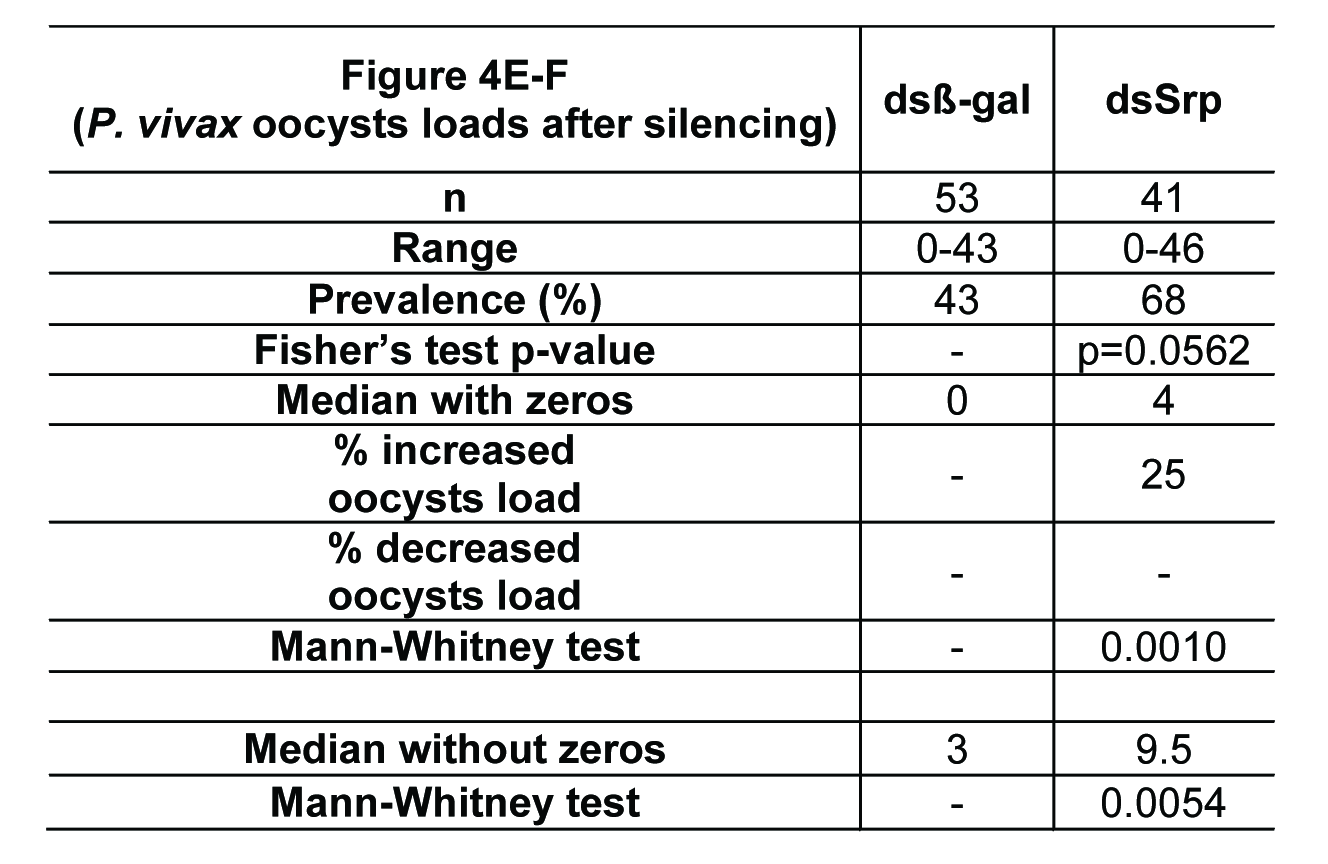

Supplement: S3 Table — P*: The blue highlighted wells indicate the significance of oocysts loads between mosquitoes injected with β-gal dsRNA (control) and Aaqu-Srp dsRNA. (TIF) [file pntd.0006785.s007.tif]
