# Supplementary material for: An Anopheles aquasalis GATA factor Serpent is required for immunity against Plasmodium and bacteria
Source: PLoS Negl Trop Dis. 2018 Sep 24;12(9):e0006785. doi: 10.1371/journal.pntd.0006785 (PMC6171954; doi:10.1371/journal.pntd.0006785)
Supplement: S1 Table — (DOCX) [file pntd.0006785.s005.docx]

| **Name in Figure 1** | **Species** | **GATA Family** | **Accession Number** |
| --- | --- | --- | --- |
| Aaeg GATA Grain | *Aedes aegypti* | Grain | XP_001658239.1 |
| Aaeg GATA Pannier |  | Pannier | AAW31748.1 |
| Aaqu GATA Serpent (Scaffold1498) | *Anopheles aquasalis* | Serpent | KY614523 |
| Aaqu GATA Pannier (Scaffold1547) |  | Pannier | KY614521 |
| Aaqu GATA Grain (Scaffold396) |  | Grain | KY614522 |
| Agam GATA Grain | *Anopheles gambiae* | Grain | AGAP004228-PA |
| Agam GATA Pannier |  | Pannier | AGAP002235 |
| Agam GATA Serpent |  | Serpent | AGAP002238-PB |
| Amel GATA Grain | *Apis mellifera* | Grain | XP_016769155.1 |
| Amel GATA Pannier |  | Pannier | XP_001121210.2 |
| Amel GATA Serpent |  | Serpent | XP_016769882.1 |
| Dmel GATA Grain | *Drosophila melanogaster* | Grain | NP_001262366.1 |
| Dmel GATA Pannier |  | Pannier | NP_001262620.1 |
| Dmel GATA Serpent |  | Serpent | NP_001247128.1 |
| Isca GATA Grain | *Ixodes scapularis* | Serpent | XP_002434087.1 |
| Nvec GATA | *Nematostela vectenis* | - | AAR24452.1 |
| Tcas GATA Grain | *Tribolium castaneum* | Grain | NP_001158260.1 |
| Tcas GATA Pannier |  | Pannier | XP_008200488.1 |
| Tcas GATA Serpent |  | Serpent | XP_008200495.1 |
